# Return to work after discharge from the intensive care unit: a Brazilian multicenter cohort

**DOI:** 10.5935/0103-507X.20220169-en

**Published:** 2022

**Authors:** Mariana F Mattioni, Camila Dietrich, Daniel Sganzerla, Régis Goulart Rosa, Cassiano Teixeira

**Affiliations:** 1 Postgraduate Program in Rehabilitation Sciences, Universidade Federal de Ciências da Saúde de Porto Alegre - Porto Alegre (RS), Brazil.; 2 Intensive Care Unit, Hospital Moinhos de Vento - Porto Alegre (RS), Brasil.; 3 Research Projects Office, Hospital Moinhos de Vento - Porto Alegre (RS), Brazil.

**Keywords:** Return to work, Health expenditures, Prognosis, Intensive care units

## Abstract

**Objective:**

To describe the rate and factors related to nonreturn to work in the third month after discharge from the intensive care unit and the impact of unemployment, loss of income and health care expenses for survivors.

**Methods:**

This was a prospective multicenter cohort study that included survivors of severe acute illness who were hospitalized between 2015 and 2018, previously employed, and who stayed more than 72 hours in the intensive care unit. Outcomes were assessed by telephone interview in the third month after discharge.

**Results:**

Of the 316 patients included in the study who had previously worked, 193 (61.1%) did not return to work within 3 months after discharge from the intensive care unit. The following factors were associated with nonreturn to work: low educational level (prevalence ratio 1.39; 95%CI 1.10 - 1.74; p = 0.006), previous employment relationship (prevalence ratio 1.32; 95%CI 1 10 - 1.58; p = 0.003), need for mechanical ventilation (prevalence ratio 1.20; 95%CI 1.01 - 1.42; p = 0.04) and physical dependence in the third month after discharge (prevalence ratio 1.27; 95%CI 1.08 - 1.48; p = 0.003). Survivors who were unable to return to work more often had reduced family income (49.7% *versus* 33.3%; p = 0.008) and increased health expenditures (66.9% *versus* 48.3%; p = 0.002). compared to those who returned to work in the third month after discharge from the intensive care unit.

**Conclusion:**

Intensive care unit survivors often do not return to work until the third month after discharge from the intensive care unit. Low educational level, formal job, need for ventilatory support and physical dependence in the third month after discharge were related to nonreturn to work. Failure to return to work was also associated with reduced family income and increased health care costs after discharge.

## INTRODUCTION

The world population is increasing and aging rapidly.^(1)^ Thus, an increasing number of patients require intensive care^(2)^ and are able to survive a severe acute illness.^(3)^ In this context of recovery, regaining the ability to return to work seems to be a logical and healthy outcome. However, survivors of a severe acute illness begin to face new physical disabilities, psychological changes and cognitive deficits^(4-7)^ that can prevent them from working again.

Approximately 40 - 65% of patients discharged from intensive care units (ICUs) were no longer working or studying before suffering a severe acute illness.^(8-13)^ Among those who worked before admission to the ICU, there is a high rate of nonreturn to work (due to retirement or dismissal) or nonreturn to usual activities of life (for example, studying), reaching 30 - 58% in 3 months after discharge,^(9,13,14)^ 30 - 49% at 6 months^(11^.^14-16)^ and 47 - 65% at 12 months.^(8-11,14,15,17-19)^ In addition, for those patients able to return to the labor market, the need to reduce working hours or change job responsibilities is frequent. In this regard, income reduction has been demonstrated both for unemployed survivors of severe acute illness^(8^.^14)^ and for those who return to the labor market after discharge from the ICU.^(14)^

Most authors who investigated the outcome return to work after ICU discharge did so as a secondary outcome,^(15^.^17-23)^ prioritizing the evaluation of quality of life as the main outcome. In addition, these studies were conducted in North American and European populations, and to date, there are no studies (to our knowledge) on return to work after severe acute illness in Brazil.

Some authors^(9^.^24)^ have already associated the nonreturn to employment with the worsening of cognition. Others^(19,21,23)^ associate it with the presence of psychological disorders, such as posttraumatic stress disorder (PTSD) or depression.^(16)^ There are still others who reported a connection with worsening health-related quality of life.^(10-12)^ The inability to return to work is believed to be a significant outcome for the patient and a consequence of the onset or worsening of these motor, psychological or cognitive deficits prevalent in ICU survivors.^(14)^

Thus, this study aimed to describe the rate and factors related to nonreturn to work in the third month after discharge from the intensive care unit, in addition to the impacts of unemployment, loss of income and health expenditures for survivors.

## METHODS

This study is a subanalysis of the Evaluation of Quality of Life after ICU Discharge study.^(25)^ This was a prospective, multicenter cohort study conducted from 2015 to 2018 in ten Brazilian medical-surgical ICUs of public or private hospitals, covering the five macroregions of the country. The study was approved by the Ethics Committee of the *Universidade de Ciências da Saúde de Porto Alegre* (No. 160,969) and by all the hospitals that participated in the data collection.

The study included patients older than 18 years, survivors of ICU admission, with a length of stay in the ICU ≥ 72 hours in cases of urgent clinical or surgical hospitalization and ≥ 120 hours in cases of elective surgical hospitalization. All signed the Free and Informed Consent Form.

Exclusion criteria were patients who were not working prior to severe acute illness, those readmitted to the ICU during the same hospital stay, those transferred directly from another hospital to the ICU, those discharged from the ICU to home or to another hospital, those in respiratory isolation after discharge from the ICU and those who did not provide informed consent or had no telephone contact information. Patients who were discharged from the ICU while still in the hospital were consecutively screened and invited to participate in the study. Consent was obtained from the patient or his or her guardian. Data on ICU admission were collected during hospitalization.

The following were evaluated: sociodemographic characteristics (age, sex, whether work was formal or informal, education, monthly family income); health status before ICU admission (presence of comorbidities, assessed by the Charlson comorbidity index,^(26)^ and physical-functional status, measured by the Barthel index);^(27)^ characteristics of severe acute illness (type of ICU admission; risk of death on ICU admission, estimated risk of death as a percentage using the *Acute* Physiology and Chronic Health Evaluation II - APACHE II or the Simplified Acute Physiology Score 3 - SAPS 3; diagnosis of sepsis, defined by the criteria for sepsis-II,^(28)^ and acute respiratory distress syndrome - ARDS, according to the Berlin definition;^(29)^ organ dysfunction during ICU stay, as a need for mechanical ventilation, vasopressors, renal replacement therapy, parenteral nutrition and transfusion of blood products and *delirium;* and length of stay in the ICU); and health status after immediate discharge from the ICU (cognitive alteration measured by the Mini-Mental State Exam - MMSE, degree of muscle strength measured by the Medical Research Council - CRM^(25)^ and presence of symptoms of anxiety and depression measured by *the* Hospital Anxiety and Depression Scale - HADS).^(25)^

All outcomes were assessed by telephone interviews in the third month after ICU discharge, which were conducted by trained researchers. The evaluation of employment *status* was performed using a direct question. To assess the change in family income and health care expenditures, the participants were asked whether the values had increased, decreased or remained unchanged compared to income and expenditures prior to hospitalization. The return to work rate at 3 months among patients working at the time of admission to the ICU was considered the primary outcome measure.

The secondary outcomes evaluated were factors related to nonreturn to work, change in family income and health-related costs, comparing the period before and after admission to the ICU.

### Statistical analysis

Categorical variables were described as absolute and relative frequencies, while continuous variables were described as the mean and standard deviation or the median and interquartile range (IQR), according to the distribution of the variable. The factors associated with nonreturn to work were assessed using modified Poisson regression models, with robust variance estimation. All variables with p < 0.20 in the univariate models were included in the multivariate model and selected according to the forward method. The results are presented as the prevalence ratio (PR) and 95% confidence interval (95%CI). The outcomes of health expenditure variation and family income were compared using Pearson’s chi-square test. The adopted significance level was 5%, and the analyses were performed using R *software*, version 3.6.0.^(30)^

## RESULTS

### Patient characteristics

**Figure 1:**
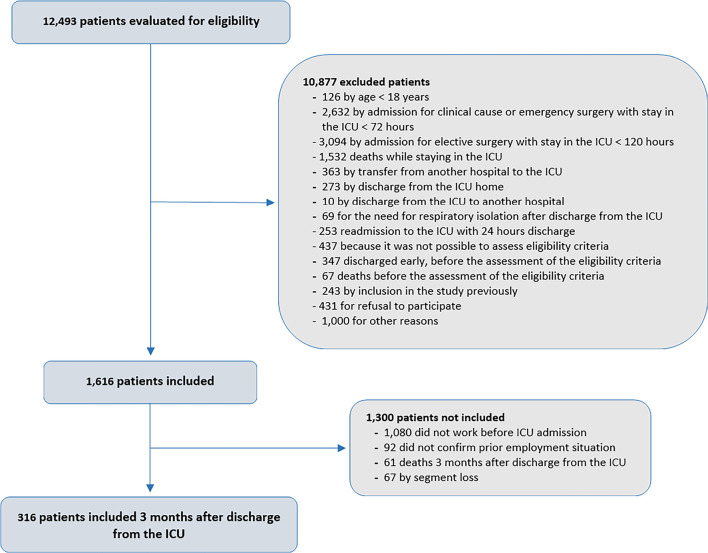
Flowchart of the study population

The demographic and clinical characteristics of the cohort, as well as the data on the severity of the acute disease, are shown in table 1. We evaluated 316 patients who were working prior to ICU admission and were alive 3 months after ICU discharge (Figure 1). The median age of the included patients was 54 years (IQR 36.8 - 63.0), 22.8% were ≥ 65 years, and 33.5% were women. The median level of education was 11 years (IQR 8 - 16). The median *per capita* family income was R$3,088 (IQR 1,793 - 7,484). Among the reasons for ICU admission, 69.3% of the patients were admitted due to medical conditions, 15.2% due to elective surgery and 15.5% due to emergency surgery. At ICU discharge, 65.5% of the patients had cognitive dysfunction, 76.1% had muscle weakness, 60.1% had symptoms of anxiety and 78.7% had symptoms of depression. In the third month after ICU discharge, 79.3% of the patients were physically dependent, and 50.5% had functional loss compared to their state prior to ICU admission.

**Table 1:** Demographic and clinical characteristics after discharge from the intensive care unit

Variables	Total (n = 316)	Did not return to work (n = 193)	Returned to work (n = 123)	Prevalence ratio (IC95%)	p value
Sociodemographic characteristics					
Age (years)	54 (36.8 - 63.0)	53 (36 - 62)	55 (40.0 - 65.5)	0.998 (0.993 - 1.003)	0.39
Age ≥ 65	72/316 (22.8)	38/193 (19.7)	34/123 (27.6)	0.83 (0.66 - 1.05)	0.13
Female sex	106/316 (33.5)	67/193 (34.7)	39/123 (31.7)	1.05 (0.88 - 1.26)	0.58
Formal work	188/313 (60.1)	123/190 (64.7)	65/123 (52.8)	1.22 (1.01 - 1.48)	0.04
Education, years	11 (8 - 16)	11 (6 - 11)	11 (10 - 16)	0.97 (0.95 - 0.99)	0.002
Low education level (no Higher Education)	233/316 (73.7)	153/193 (79.3)	80/123 (65.0)	1.36 (1.07 - 1.73)	0.01
Monthly household income per capita, R$	3,088 (1793 - 7484)	2,470.5 (1,431 - 5,753)	4,940.5 (2,446 - 9,881.5)	1.00 (0.99 - 1.01)	0.29
Health status before ICU admission					
Charlson comorbidity index	1 (0 - 2)	1 (0 - 2)	0 (0 - 2)	1.03 (1.00 - 1.07)	0.04
Charlson comorbidity index ≥ 2	119/316 (37.7)	77/193 (39.9)	42/123 (34.1)	1.10 (0.92 - 1.32)	0.28
History of dementia	2/316 (0.6)	2/193 (1.0)	0/123 (0)	-	-
History of depression	36/313 (11.5)	23/190 (12.1)	13/123 (10.6)	1.06 (0.81 - 1.38)	0.67
History of anxiety	49/313 (15.7)	31/190 (16.3)	18/123 (14.6)	1.05 (0.83 - 1.33)	0.68
Barthel index	100 (100 - 100)	100 (100 -100)	100 (100 - 100)	1.011 (0.955 - 1.071)	0.70
Physical independence	261/315 (82.9)	154/193 (79.8)	107/122 (87.7)		
Mild physical dependence	45/315 (14.3)	34/193 (17.6)	11/122 (9.0)		
Moderate physical dependence	6/315 (1.9)	4/193 (2.1)	2/122 (1.6)		
Severe physical dependence	1/315 (0.3)	1/193 (0.5)	0/122 (0.0)		
Total physical dependence	2/315 (0.6)	0/193 (0.0)	2/122 (1.6)		
Moderate/severe physical dependence (Barthel < 75)	9/315 (2.9)	5/193 (2.6)	4/122 (3.3)	0.90 (0.50 - 1.63)	0.74
Features of severe acute illness					
Type of ICU admission					
Clinic	219/316 (69.3)	128/193 (66.3)	91/123 (74.0)	0.93 (0.73 - 1.19)	
Surgical, elective	48/316 (15.2)	30/193 (15.5)	18/123 (14.6)	Reference	
Surgical, emergency	49/316 (15.5)	35/193 (18.1)	14/123 (11.4)	1.14 (0.86 - 1.51)	
Risk of death on ICU admission, %	14.6 (8.7 - 26.2)	16.5 (9.9 - 29.1)	12.9 (8.7 - 18.8)	1.006 (1.002 - 1.009)	0.002
Sepsis	84/316 (26.6)	52/193 (26.9)	31/123 (26.0)	1.02 (0.84 - 1.24)	0.85
ARDS	24/316 (7.6)	12/193 (6.2)	12/123 (9.8)	0.81 (0.54 - 1.22)	0.31
Organ dysfunction during ICU stay					
Number of organic dysfunctions	1 (0 - 2)	1 (0 - 3)	1 (0 - 2)	1.10 (1.04 - 1.16)	0.001
Need for VM	153/316 (48.4)	106/193 (54.9)	47/123 (38.2)	1.29 (1.08 - 1.55)	0.005
Need for vasopressor	145/316 (45.9)	95/193 (49.2)	50/123 (40.7)	1.14 (0.96 - 1.36)	0.13
Need for RRT	41/316 (13.0)	29/193 (15.0)	12/123 (9.5)	1.19 (0.95 - 1.48)	0.13
Need for parenteral nutrition	16/316 (5.1)	14/193 (7.3)	2/123 (1.5)	1.47 (1.19 - 1.80)	0.001
Need for transfusion	56/316 (17.7)	40/193 (20.7)	16/123 (13.0)	1.22 (1.01 - 1.48)	0.05
*Delirium*	64/316 (20.3)	43/193 (22.3)	21/123 (17.1)	1.13 (0.92 - 1.38)	0.23
Infection acquired in the ICU	52/316 (16.5)	42/193 (21.8)	10/123 (8.1)	1.41 (1.19 - 1.67)	< 0.001
Length of stay in the ICU	6 (4.0 - 11.2)	7 (5 - 14)	6 (4 - 9)	1.014 (1.010 - 1.019)	< 0.001
Length of hospital stay	23 (14 - 38)	30 (18 - 53)	15 (11.0 - 22.5)	1.008 (1.006 - 1.010)	< 0.001
Health status immediately after ICU discharge (24 to 120 hours)					
Cognitive dysfunction	150/229 (65.5)	78/133 (58.6)	72/96 (75.0)	1.34 (1.08 - 1.65)	0.007
Muscle weakness (MRC < 48)	159/209 (76.1)	83/120 (69.2)	76/89 (85.4)	1.42 (1.14 -1.77)	0.002
Anxiety symptom (HADSa > 7)	161/268 (60.1)	84/152 (55.3)	77/116 (66.4)	1.22 (0.99 - 1.50)	0.06
Symptom of depression (HADSd >7)	211/268 (78.7)	115/152 (75.7)	96/116 (82.8)	1.19 (0.95 - 1.49)	0.13
After 3 months of ICU discharge					
Physical dependence (Barthel < 75)	241/304 (79.3)	127/182 (69.8)	114/122 (93.4)	1.66 (1.42 - 1.93)	< 0.001
Functional loss (Barthel drop > 5 points)	153/303 (50.5)	121/182 (66.5)	32/122 (26.2)	1.94 (1.58 - 2.40)	< 0.001

95%CI - 95% confidence interval; ICU - intensive care unit; ARDS - acute respiratory distress syndrome; MV - mechanical ventilation; RRT - renal replacement therapy; MRC - Medical Research Council; HADS - Hospital Anxiety and Depression Scale. The results are expressed as the median (interquartile range) or total n/n (%).

ICU - intensive care unit.

### Probability of not returning to work

**Figure 2:**
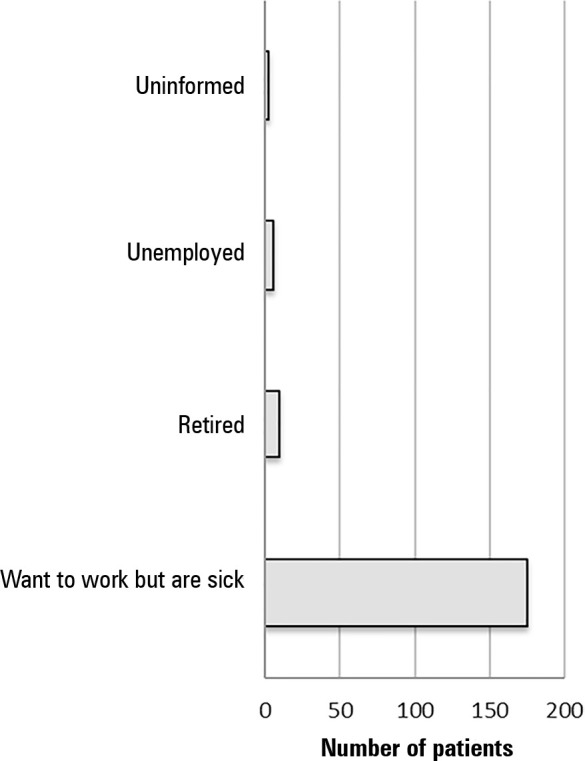
Reasons for not returning to work 3 months after discharge from the intensive care unit.

**Figure 3:**
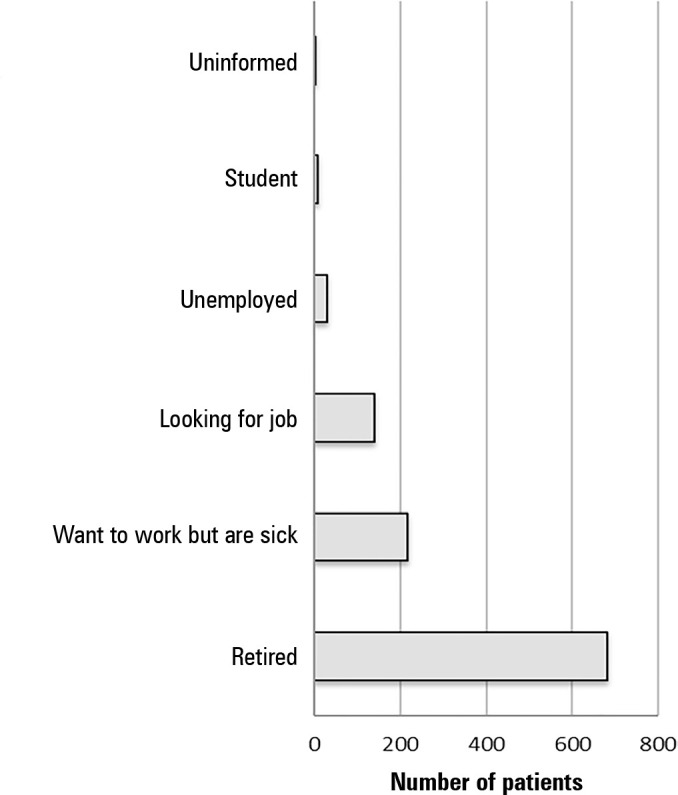
Reasons not to work prior to admission to the intensive care unit.

Of 316 patients employed prior to hospitalization, 193 (61.1%) did not return to work in the 3 months after ICU discharge. Figure 2 shows the main reasons why those who worked prior to ICU admission did not return to work in the 3 months following discharge. Leave due to health problems (current sick leave certificate provided by the Unified Health System - SUS) was responsible for 91.6% of the cases of nonreturn to work within 3 months. The remaining small percentage were not working because they retired during that period or lost their jobs. In addition, retirement was the main reason for not working prior to admission to the ICU, and absence from work for health reasons was responsible for approximately 20% of absences (Figure 3).

ICU - intensive care unit.

ICU - intensive care unit.

### Factors associated with nonreturn to work

Multivariate analysis (Table 2) showed that the factors associated with nonreturn to work after ICU discharge were low educational level (PR 1.39; 95%CI 1.10 - 1.74; p = 0.006), having a formal job (PR 1.32; 95%CI 1.10 - 1.58; p = 0.003), need for mechanical ventilation (PR 1.20; 95%CI 1.01 - 1.42; p = 0.04) and physical dependence in the third month (RP 1.27; 95%CI 1.08 - 1.48; p = 0.003).

**Table 2:** Multivariate analysis of factors related to nonreturn to work

Variable	Events/exhibited	Events/not exposed	RP (IC95%)	p value
Low education level (%)	153/233 (65.7)	40/83 (48.2)	1.39 (1.10 - 1.74)	0.006
Need for mechanical ventilation (%)	106/153 (69.3)	87/143 (60.8)	1.20 (1.01 - 1.42)	0.039
Formal employment (%)	123/188 (65.4)	67/125 (53.6)	1.32 (1.10 - 1.58)	0.003
Physical dependence in the 3rd month (%)	55/63 (87.3)	127/241 (52.7)	1.27 (1.08 - 1.48)	0.003

RP - razão de prevalência; IC95% - intervalo de confiança de 95%.

### Variation in family income and health expenditures

Survivors who were unable to return to work more often had reduced family income (49.7% *versus* 33.3%; p = 0.008) and increased health expenditures (66.9% *versus* 48.3%; p = 0.002) compared to those who returned to work in the third month after ICU discharge.

## DISCUSSION

The study data showed that 61.1% of critically ill patients were unable to return to work in the first 3 months after ICU discharge. The risk of nonreturn is related to prehospitalization factors (low educational level and having a formal job), disease severity (requirement of mechanical ventilation during ICU stay) and motor sequelae after discharge (physical dependence). In addition, this subgroup of patients reported higher health care expenditures and reduced family income.

Previous data have shown that 40% to 65% of critically ill patients admitted to ICUs no longer work prior to admission.^(8-13)^ This is probably related to the greater aging of the population and the high prevalence of comorbidities found in these patients.^(1^.^31)^ Regarding workers, a recent systematic review with meta-analysis (52 studies with 10,015 patients)^(14)^ showed that only 36% (23% - 49%) of survivors were able to return to work within 3 months of ICU discharge.

A negative impact in the ability to work was evident in patients discharged from the ICU.^(8-11,15,17-24)^ Individuals who remain in the labor market may experience difficulties such as underemployment, the need to reduce working hours, transition to a part-time job or to a less important position, or even obtain sick leave.^(22)^ In addition, many patients receive a disability pension in the first months after discharge from the ICU.^(22)^ The data evidenced in this article identified that health problems were the reason for absence from work in more than 90% of the cases, a fact that may have led to a high rate of disability retirement in the following months, complying with the current norms of Brazilian legislation.

Regarding the evaluation of risk factors for nonreturn to work, robust studies have identified the following related causes:^(14)^ low level of education, presence of comorbidities and loss of mental health after discharge, as well as hospital discharge to care clinics, indicating a higher degree of functional dependence. The severity of critical illness seems to lose importance when compared to prehospital admission factors with the exception of the need for invasive ventilatory support. Riddersholm et al.^(32)^ had previously associated the need for ventilatory support (hazard ratio 0.70; 95%CI 0.65 - 0.77) with a lower chance of returning to work. In the present study, a similar relationship was found (PR 1.20; 95%CI 1.01 - 1.42). This finding may be related to the fact that prolonged dependence on mechanical ventilation appears only as a surrogate indicator of muscle weakness, and its importance in the prediction is veiled by the presence of muscle weakness and functional dependence after ICU discharge.^(4,33,34)^

Several studies have also suggested an association between job loss and the presence of psychiatric symptoms.^(10,17,20,22,23,35,36)^ In the present study, this correlation was not found; however, it seems plausible that it may occur. It is noteworthy that these data were collected only at the time of discharge from the ICU and not in the third month after discharge. In addition, there was a greater return to work for patients who were self-employed before hospitalization, which is a curious finding not reported in previous studies. The reason for this difference is uncertain and may be related to the type of work (for example, manual *versus* intellectual), socioeconomic *status*, quality of care during hospitalization and/or access to post-ICU rehabilitation services.

The strength of this study includes a multicenter design, including the five regions of the country, as well as public and private hospitals. This is also the first Brazilian study designed to evaluate return to work after the ICU, with a robust sample size. However, the study has some limitations. First, the family income data were reported by the patient, which may be influenced by external factors (economic crisis in the country or inflation) and internal factors (embarrassment in sharing these data and fear of losing possible financial benefits from government agencies). Additionally, because this was an observational cohort study, causality between factors related to severe acute illness and nonreturn to work could not be defined.

## CONCLUSION

More than half of critically ill patients are unable to return to work in the first 3 months after discharge from the intensive care unit. This risk is related to pre-intensive care unit factors (low educational level and having a formal job), the severity of the acute disease (need for ventilatory support) and physical limitations after discharge. These findings support the importance of rehabilitation in order to minimize sequelae after severe acute illness and facilitate the return to work.
